# Bufotenine, a tryptophan-derived alkaloid, suppresses the symptoms and increases the survival rate of rabies-infected mice: the development of a pharmacological approach for rabies treatment

**DOI:** 10.1590/1678-9199-JVATITD-2019-0050

**Published:** 2020-02-03

**Authors:** Hugo Vigerelli, Juliana M. Sciani, Patricia M. C. Pereira, Aline A. Lavezo, Andrea C. R. Silva, Rita C. O. Collaço, Thalita Rocha, Thais C. Bueno, Daniel C. Pimenta

**Affiliations:** 1 Laboratory of Biochemistry and Biophysics, Butantan Institute, São Paulo, SP, Brazil.; 2 Laboratory of Biological Quality Control in vivo, Butantan Institute, São Paulo, SP, Brazil.; 3 Laboratory of Rabies Diagnostic, Serology, Pasteur Institute, São Paulo, Brazil; 4 Department of Pharmacology, State University of Campinas (Unicamp), Campinas, SP, Brazil.; 5 Multidisciplinary Research Laboratory, São Francisco University, Bragança Paulista, SP, Brazil.

**Keywords:** Rabies, Rabies therapy, Bufotenine

## Abstract

**Background:**

Between 40,000-70,000 people die yearly of rabies, an incurable disease. Besides post-bite vaccination, no treatment is available for it.

**Methods:**

First, virus dilution for antiviral effects in mice was determined. Then, animals were treated as follows: control (NaCl 250 µL/animal/day); bufotenine (0.63, 1.05 and 2.1 mg in 250 µL of NaCl/animal/day); rabies (10^-6,82^CVS dilution); and test (10^-6,82^ CVS dilution and bufotenine, in the above-mentioned doses). Animals were observed daily for 21 days or until the 3^rd^ stage of rabies infection. Twitch-tension and liposome studies were applied to understand the possible interaction of bufotenine with receptors, particularly acetylcholine.

**Results:**

Bufotenine was able to increase the survival rate of intracerebrally virus-infected mice from 15 to 40%. Bufotenine did not seem to interfere with the acetylcholine response in the skeletal muscle, indicating that its mechanism of action is not blocking the virus entrance due to nAChR antagonism. By analyzing liposomes, we could observe that bufotenine did not passively penetrates cell membranes, indicating the necessity of complementary structures to cell penetration.

**Conclusions:**

Bufotenine is a promising candidate for drug development. After further chemical modification, it might be possible to dissociate minor side effects, increase efficiency, efficacy and pharmacokinetics, yielding a true anti-rabies drug.

## Background

Rabies is a disease that occurs in more than 150 countries, where 3 billion people are exposed to infection and 40,000-70,000 people die every year [1-3]. It is a 100% vaccine-preventable disease and in over 99% of the cases, the virus is transmitted to humans by domestic dogs [1]. The WHO fact-sheet on rabies states that “Globally, rabies deaths are rarely reported and children between the ages of 5-14 years are frequent victims. Treating a rabies exposure, where the average cost of rabies post-exposure prophylaxis is US$ 40 in Africa, and US$ 49 in Asia, can be a catastrophic financial burden on affected families whose average daily income is around US$ 1-2 per person”.

There are only ten reported cases of rabies cure in the literature that do not lack scientific evidence, and only three of them had completely recovered or suffered mild sequelae [4-14]. There is constant research on new, more effective and less expensive agents displaying potential antiviral activities on rabies [15-17]. However, in spite of the knowledge gathered on aspects of the disease and the host immune response, no antiviral compound with reproducible activity in animal models for rabies has been found [18].

Taking this into account, we have performed the bio-monitored screening of the amphibian skin secretion targeting anti-rabies molecules, based on that one possible mechanism of the virus penetration in mammal cells is via the nicotinic acetylcholine receptor (nAChR) [19], and some amphibians secrete a myriad of alkaloids in their skin, which would bind these receptors [20]. We have successfully described that bufotenine inhibits rabies virus infection in mammalian cells in a dose and time-dependent manner [21]. Moreover, bufotenine acted synergistically with a synthetic tetrapeptide derived from the natural ocellatin-F1 [22], similar to the rabies virus glycoprotein region associated with the viral cell penetration [23]. As bufotenine’s actual mechanism of action is still under investigation, the safety of the chronic administration on mice was assessed [24]. Here, behavioral and biological aspects were observed, in order to study the bufotenine as a possible interfering agent in the process of rabies virus infection *in vivo*.

## Methods

### Ethics statement

All *in vivo* experiments were approved by the Ethics Committee on Animal Use of the Butantan Institute (CEUAIB - 9532050216).

### Reagents

All reagents were of analytical grade and were purchased from Sigma Aldrich (USA), unless otherwise stated.

### Bufotenine

*Anadenanthera colubrina* seeds were obtained from the legitimate supplier Arbocenter Comércio de Sementes Ltda, Birigui, São Paulo, Brazil (batch 0019). Bufotenine was purified as previously described [21].

### Mice, cells and viruses

Swiss mice (*Mus musculus*), both male and female (21 g) were employed in antiviral experiments and male mice (30 g), in the twitch-tension studies. Animals were housed (3-6 per cage), at room temperature (22 ± 2^o^C) and 12:12 h light:dark cycle, with free access to food and water.

Mouse neuroblastoma (N2A) cell line (ATCC® CCL) were cultured in MEM-10 medium, with Earle’s balanced salts and supplementation of essential amino acids, at 37^o^C, in a humidified 5% CO_2_ atmosphere, until the formation of the cell monolayer.

Three strains of street rabies viruses circulating in Brazil were isolated from central nervous system samples of naturally infected animals (a dog, the insectivorous bat *Eptesicus furinalis* and a bovine that was infected by the hematophagous bat *Desmodus rotundus*) positively diagnosed for such strains through genome sequencing performed in the Virology Laboratory, Pasteur Institute, São Paulo, Brazil [25]. *Rabies virus* challenge virus standard (CVS/31 batch 31/3#2/IB-6) were also used to determine the antiviral activity.

### Cytotoxicity of bufotenine in N2A cells

The cytotoxicity evaluation of bufotenine was performed by the MTT [3-(4,5-dimethylthiazol-2-yl)-2,5-diphenyltetrazolium bromide] method, according to Takeuchi et al. [26] and Mosmann [27], with slight modifications. Briefly, N2A cells (5 × 10^5^ cells/well) were deposited in 96-well microtiter plates and incubated with 50 μL (constant volume) of different bufotenine concentrations (from 1 to 8 mg/mL) diluted in MEM-10. Negative control was MEM-10. After 96 hours at 37^o^C, under a humidified 5% CO_2_ atmosphere, the medium was removed and 50 μL 1 mg.mL^-1^ MTT, in MEM-10, was added, following a four-hour incubation period. Next, the MTT solution was removed and 100 μL dimethyl sulfoxide (DMSO) was added to each well. After gentle shaking of the plates, the absorbance was measured (Molecular Devices®, SpectraMax M2) at 540 nm. The cytotoxic concentration (CC_50_) value was defined as bufotenine concentration able to reduce the MTT assay absorbance of treated cells by 50%, when compared to the control, untreated cells.

### N2A cells fluorescence inhibition test

This test was performed according to a previous study [28], with modifications. Briefly, different rabies virus strains suspensions were deposited on 96-well microtiter plates that were kept on ice bath during sample preparation. Then, 110 µL MEM-10 and 50 µL bufotenine (1.5 or 0.75 mg/mL) were added. Samples were homogenized and, following removal from ice, 100 µL of N2A cells (5 x 10^5^) were added. After a 96-hour incubation period at 37^o^C, under a humidified 5% CO_2_ atmosphere, the supernatants were collected for subsequent virus titration. Microplates were returned to ice. Cells were fixed with cold 80% acetone addition. After 15 minutes, the microplates were emptied by inversion and dried at 37^o^C [29, 30]. 

Viral presence in the cultured was immunologically evaluated - 40 µL of an optimal dilution antirabies fluorescent conjugate was incubated for 1h [31]. Later, the plates were washed with PBS and viewed under fluorescence microscopy (Leica DMIL, 100x magnification). Infection inhibition was determined according to the fluorescence intensity displayed by the cell monolayer, in comparison to the negative control (no bufotenine). The supernatant virus titers were determined by plate assay [29, 30] adapted to N2A cells and expressed as-log TCID 50/mL, using the Spearman-Karber analysis.

### *In vivo* experiments

The *in vivo* experiments, according to the WHO procedures for rabies (observation of infected animals for 21 consecutive days and monitoring of symptoms development and survival rate) [32] were divided into two cycles; and each and all experimental groups contained ten animals. Cycle 1 was necessary to establish the best virus dilution to evaluate antiviral effects. It consisted of the following treatments:

- control group: subcutaneous (SC) inoculation of NaCl 0.9 %, 250 µL/animal/day;
- bufotenine group: SC inoculation of 0.63 mg bufotenine, in 250 µL NaCl/animal/day;
- rabies group: (intracerebral inoculation route) for four dilutions of the CVS virus - 10^-5.32^ (corresponding to the challenge dose of approximately 30LD_50_), 10^-6.32^ (corresponding to 3LD_50_), 10^-6.82^ (corresponding to 1.35LD_50_) and 10^-7.32^ (corresponding to 0.3LD_50_);
- bufotenine-treated group: mice inoculated with the same four dilutions of the CVS virus and treated with bufotenine (0.63 mg/250 µL NaCl).

Cycle 2, on the other hand, was necessary to establish the best bufotenine dose to evaluate the antiviral effects. It consisted of the following treatments:

- control group: SC inoculation of NaCl 250 µL/animal/day;
- bufotenine group: subcutaneous inoculation of 0.63, 1.05 and 2.1 mg in 250 µL of NaCl/animal/day;
- rabies group: intracerebral inoculation route with CVS dilution of 10^-6,82^;
- treatment group: mice inoculated with the same CVS dilution and treated with each bufotenine dose.

The progression evaluation of infected animals’ symptoms was divided into three stages. The first stage consisted onset of hunchback, prostration and piloerection. Although these signs are not specific to rabies, they indicate that the animal is ill and that its welfare is compromised. In the second stage the animal shows slow or circular movements, towards a single direction. These are the first clinical indicators of neurological disorders. In the third stage, the animal presents unstable movements, tremors and/or convulsions. These symptoms can be aroused when provoking the animal with tweezers. These neurological signs clearly indicate rabies infection. Animals were observed daily, for 21 days, or until the development of the third stage of rabies, when the animals were euthanized, avoiding the slow, progressive and irreversible animal suffering caused by the infection.

### Experiments with liposomes

Liposomes were obtained and analyzed as described by Sciani et al. [33]. Briefly, liposomes (10 µL) and bufotenine (10 µg/10 µL) were separately analyzed for retention time determination. Next, bufotenine and liposomes were incubated (20 µL, 1:1 V/V) for 10 minutes at room temperature, and the mixture was analyzed under the same conditions. The liposome peak was collected and lysed with 50% acetonitrile containing 0.1% formic acid. This solution was then sonicated and centrifuged for 10 minutes at 10840 x g at 4°C. The supernatant was analyzed by electrospray-ion trap-time of flight (ESI-IT-TOF) (Shimadzu Co., Japan) equipped with binary ultra-fast liquid chromatography system (UFLC) (20A Prominence, Shimadzu Co., Japan), in order to identify bufotenine inside the liposomes.

### Phrenic nerve-diaphragm muscle (PND) preparation

Mice were euthanized with an overdose of isoflurane (via inhalation). The hemidiaphragms and corresponding phrenic nerves were carefully removed and mounted under a tension of 5 g in 5 mL organ baths containing Tyrode solution (composition in nM: NaCl 137, KCl 2.7, CaCl_2_ 1.8, MgCl_2_ 0.49, NaH_2_PO_4_ 0.42, NaHCO_3_ 11.9 and glucose 11.1) gassed with 95% O_2_ and 5% CO_2_ at 37^o^C. The muscles were indirectly stimulated (nerve-evoked contractions; 0.1 Hz, 0.2 ms, supramaximal voltage; Grass S48 stimulator). The resulting muscle tension was recorded using a force displacement transducer (BG 25 GM Kulite) coupled to a Gould RS 3400 recorder. The preparations were allowed to stabilize for at least 15 minutes before the addition of bufotenine (21 and 210 μg/mL).

### Morphological analysis

After 120 minutes of bufotenine incubation, diaphragm muscles were fixed in 10% formaldehyde overnight, dehydrated in graded ethanol (70%, 80%, 95% and 100%), cleared in xylene and embedded in paraffin. Transversal sections (5-μm thick) were mounted on glass slides for hematoxylin-eosin (HE) staining for histological analyses. The slides were examined with a Nikon Eclipse E200 light microscope (Nikon, Japan) and the images were captured and qualitatively analyzed using NIS Elements 4.60.000 AR software.

### Statistical analysis

All the results are expressed as mean ± SEM. The CC_50_ of bufotenine concentration for N2A cells was calculated from concentration-effect curves, by nonlinear regression analysis, of two independent experiments, performed in triplicates. Survival curves were compared using Log-rank (Mantel-Cox) Test and/or the Gehan-Breslow-Wilcoxon Test, which attributes higher statistical weight to experimental points corresponding to deaths occurring at early time points [34]. The twitch-tension responses were expressed as a percentage of the basal values of each preparation, taken as 100% prior to the addition of bufotenine. Statistical comparisons were done using analysis of variance (ANOVA) followed by the Bonferroni test. All data analyses were done using Prism (GraphPad Inc., La Jolla, USA) and the significance set as: ns p > 0.05, * p ≤ 0.05, ** p ≤ 0.01.

## Results

### N2A cells fluorescence inhibition test

**Figure 1:**
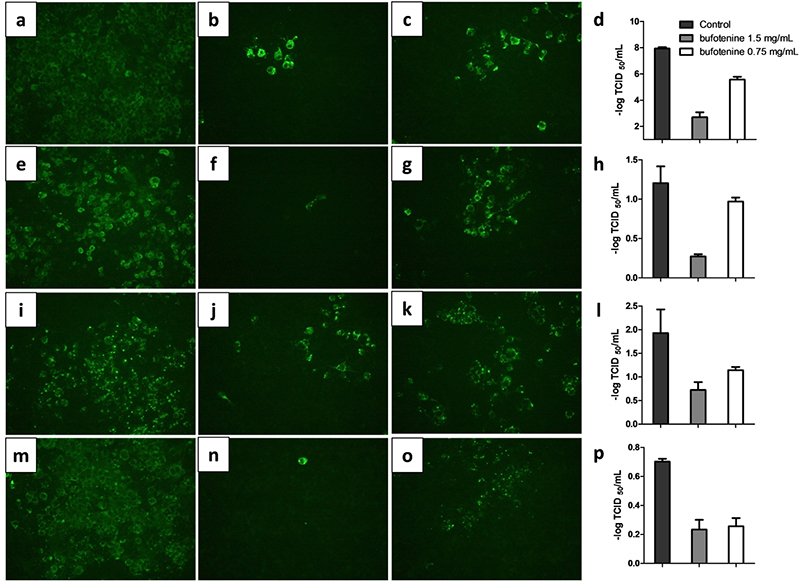
Bufotenine inhibits different RABV strains in N2A cells. Inhibition of challenge virus standard (CVS): **(a)** control; **(b)** bufotenine concentration of 1,5 mg/mL; **(c)** bufotenine concentration of 0.75 mg/mL; **(d)** supernatant virus titration. Inhibition of dog lineage RABV isolated from dog central nervous system sample: **(e)** control; **(f)** bufotenine concentration of 1.5 mg/mL; **(g)** bufotenine concentration of 0.75 mg/mL; **(h)** supernatant virus titration. Inhibition of *Eptesicus furinalis* lineage RABV isolated from bat central nervous system sample: **(i)** control; **(j)** bufotenine concentration of 1.5 mg/mL; **(k)** bufotenine concentration of 0.75 mg/mL; **(l)** supernatant virus titration. Inhibition of *Desmodus rotundus* lineage RABV isolated from bovine central nervous system sample: **(m)** control; **(n)** bufotenine concentration of 1.5 mg/mL; **(o)** bufotenine concentration of 0.75 mg/mL; **(p)** supernatant virus titration.

Our results show an evident infection inhibition effect against the CVS and the wild viruses (Fig. 1) and minute cytotoxic effects for the assayed concentrations (1.5 and 0.75 mg/mL; below the CC_50_, Additional file 1), indicating that either virus strain is susceptible to the bufotenine infection inhibitory effect in mouse neuroblastoma (N2A) cell line.

### *In vivo* experiments

For the standardization and optimization of *in vivo* experiments, we have initially determined the CVS challenge dose to be 10^-5.32^ (corresponding to 30LD_50_), able to kill 100% of the infected mice (Additional file 2a and 2d).

Then, bufotenine was administered SC 0.63 mg/animal/day, according to Fuller et al. [35] who established its toxicity to be 30 mg/kg for the murine (rat) equivalent. Considering the average mouse blood volume of 80 mL.kg^-1^, 0.63 mg/animal would correspond to 0.38 mg.mL^-1^ (blood), which is ≈4 times lower than the IC_50_ previously determined (1.57 mg.mL^-1^) for BHK-21 cell virus infection inhibition [21].

**Figure 2:**
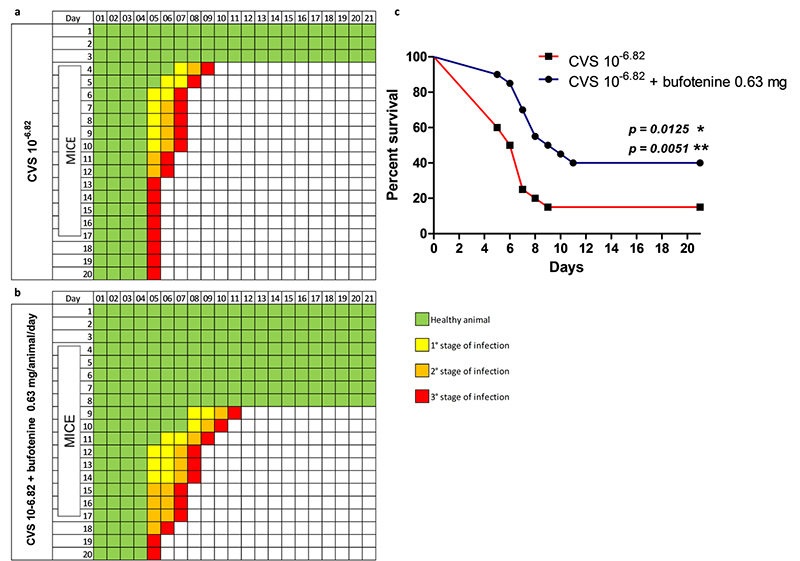
Onset of symptoms and percent survival of mice infected with CVS 10^-6.22^ (approximately 1.35LD_50_) and treated with bufotenine 0.63 mg/animal/day. **(a)** Rabies control group: symptoms onset on mice infected with CVS 10^-6.82^; **(b)** treatment group: symptoms onset on mice infected with CVS 10^-6.82^ and treated with bufotenine 0.63 mg/animal/day; **(c)** percentage survival of control group, rabies control group and treatment group. p value summary: * p ≤ 0.05 in Log-rank (Mantel-Cox) Test, ** p ≤ 0.01 in Gehan-Breslow-Wilcoxon Test.

Additionally, 10^-6,32^, 10^-6,82^ and 10^-7,32^ CVS were assayed in order to evaluate proper virus dilution and antiviral effects (Additional files 2, 3, 4 and 5). The 10^-6.82^ dilution, able to kill 80-90% of the animals, was selected. Then, two more bufotenine doses were tested at this virus dilution: 1.05 and 2.1 mg/animal/day (equivalent to 50 and 100 mg/kg, or 0.63 and 1.25 mg.mL^-1^) (Additional file 6, controls shown in Additional file 7). After all, mice infected with CVS 10^-6,82^ and treated with 0.63 mg/animal/day proved to be the best model for the antiviral effect evaluation (Fig. 2). Therefore, data reported hereafter derive from such conditions (data from both experiments).

### Experiments with liposomes

**Figure 3:**
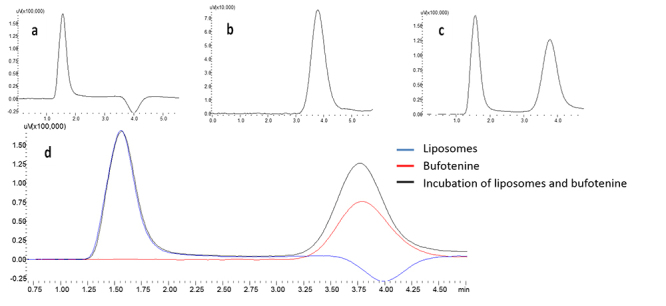
Analysis of bufotenine penetration in liposomes by size exclusion chromatography. **(a)** Positively charged liposome; **(b)** bufotenine; **(c)** incubation of liposome and bufotenine indicating no liposome penetration; **(d)** overlap of **(a), (b)** and **(c)**.

Our experiments showed that bufotenine cannot passively penetrate either positively or negatively charged liposomes, as shown in Figure 3. This data indicates that other cell structures, such as proteins, ion channels, receptors, or even endocytosis are important for bufotenine entrance into the cell.

### Phrenic nerve-diaphragm muscle (PND) and morphological analysis

**Figure 4:**
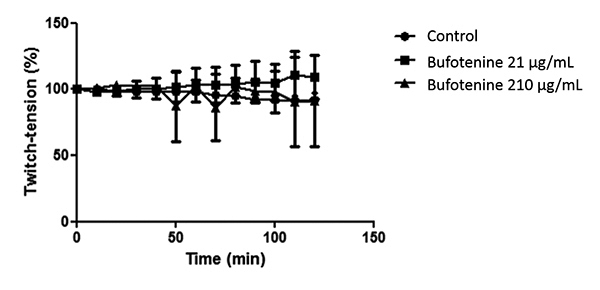
Representative neuromuscular response of phrenic nerve-diaphragm muscle (PND) preparation under stimulation. Bufotenine (21 and 210 μg/mL) was unable to induce neuromuscular blockade. The points represent the mean ± SEM of 7-4 experiments.

**Figure 5:**
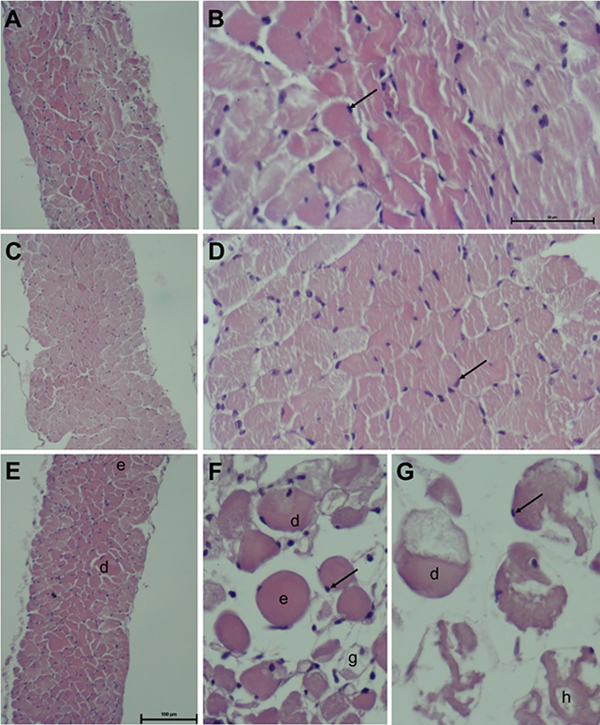
Histological analysis of diaphragm muscles incubated with Tyrode alone and bufotenine. **(A, B)** Tyrode alone, **(C, D)** 21 μg/mL of bufotenine and **(E-G)** 210 μg/mL of butotenine. Observe the normal muscle morphology in Tyrode and bufotenine at lower concentrations. However, myonecrosis can occur at 210 μg/mL of butotenine, as observed in **(F)** and **(G)**. d: delta lesion, e: edematous fiber, h: hypercontracted fiber, g: ghost fiber. Magnification: **(A, C, E)** = 100x; **(B, D, F, G)** = 400x.

In PND preparations, bufotenine (21 and 210 μg/mL) did not interfere in the contractions until 120 min analysis (Fig. 4). Light microscopy of control muscles (incubated for 120 min in Tyrode solution; Fig. 5A and 5B) and bufotenine-incubated muscles (21 μg/mL; Fig. 5C and 5D) displayed normal morphology: hexagonal fibers, with peripheric nuclei, intact sarcolemma and myofilaments distribution. These results indicated that the alkaloid caused no damage to the tissue, nor to the neuromuscular junction. However, PND preparations incubated with bufotenine at a higher concentration (210 μg/mL, Fig. 5E-G), displayed few areas of myonecrosis, with fibers in different pathologic states, including edematous, hypercontracted, delta and ghost fibers, among normal fibers, although the neuromuscular junction was also unaffected.

## Discussion

Currently, rabies laboratory research is performed with different strains of rabies viruses (RABV), namely laboratory-adapted (fixed) strains (e.g. PV and CVS) and wild-type viruses. Although fixed viruses possess the advantage of having a well-defined incubation period and predictable clinical course, these strains may have lost some of the wild-type ancestors features, such as local replication [36]. Taking this into account, we opted to use three Brazilian wild type RABV strains, first on cultured cells before the actual *in vivo* experiments, in order to confirm our previous results with the PV strain [21]. 

Interestingly, on the 5^th^ day of bufotenine *in vivo* treatment, there were still 11 healthy animals, whereas in the rabies group, there were only five (Fig. 2). Such remarkable difference was also observed for animals already displaying different stages of infection. For example, there were only two animals displaying 3^rd^ stage infection symptoms in the bufotenine-treated group, whilst eight animals in the rabies group had reached this phase at this same day. Furthermore, it was possible to observe major differences on the survival percent of the groups. Specifically, the bufotenine-treated group presented 90% survival rate, versus 60% of the rabies group on the 5^th^ day, 85% vs 50% on the 6^th^ day, 70% vs 25% on the 7^th^ day, 55% vs 20% on the 8^th^ day, 50% vs 15% on the 9^th^ day and 40% vs 15% on the 11^th^ day, which lasted until the 21^st^ and last day of experiment.

As previously observed [24], animals treated with bufotenine (and no virus) presented little histological alteration at the inoculation site and no alteration in the collected internal organs, in terms of inflammation or necrosis.

Willoughby et al. [9] reported a human survival case of a patient who developed rabies after exposure to a rabid bat, in the USA. This was the first reported survival of a patient that had not received rabies prophylaxis prior to the onset of the disease, known as the Milwaukee protocol, based on induced therapeutic coma, ketamine (anti-excitatory) and unspecific antiviral drug administration (such as ribavirin and amantadine), concomitant to patient’s passive/active immunization. The patient evolved to an almost complete recovery, including extensive physical and neurological rehabilitation [9]. However, several attempts to reproduce this treatment failed [37-39].

Zeiler and Jackson [40] questioned the efficiency and efficacy of this protocol by comparing the actual number of successful cases versus the detailed communications on the failed attempts (30+). Moreover, these authors pity the lack of therapeutic alternatives and hope for the development of new strategies based on consolidated animal models that, according to our perspective, is what we have performed in the present study.

In order to glimpse possible bufotenine mechanisms of action, we have synthesized both positively - mimicking normal healthy cells - and negatively charged liposomes. Our experiments showed that bufotenine cannot passively penetrate either liposomes or other surface molecules, suggesting that cell receptors are necessary to facilitate bufotenine cellular entry. Cell receptors have already been described to play an important role in RABV entry, cell tropism, and spread. The neuromuscular junction acetylcholine receptor (nAChR) was the first described receptor for the RABV [41]. Later, *in vitro* studies showed that neural cell adhesion molecule (NCAM) and p75 neurotrophin could be involved in the virus cellular penetration [42, 43]. Thus, we have initially postulated that bufotenine would inhibit RABV penetration through competition with nAChR [21], and then we tested the alkaloid in PND preparation, which is mainly based on acetylcholine mediation.

Interestingly, we observed that the bufotenine does not directly interact with either nAChR or mAChR, as no modifications in the muscular contraction could be detected under the employed experimental conditions. On the other hand, membrane proteins would be essential, according to Broughan and Wunner [44] that showed the importance of such proteins in the virus entrance into BHK-21, as well as our liposomes results demonstrated the incapacity of the bufotenine passive membrane penetration.

Furthermore, other cell-surface molecules, including sialic acid, galactose, mannose, N-acetylglucosamine and gangliosides, have already been demonstrated to be involved in rabies virus binding to the host cells [45, 46]. Thus, the cellular action of bufotenine remains under investigation.

## Conclusion

No specific effective rabies antiviral compound does exist [18]. Bufotenine presents a novel pharmaceutical prototype to be further developed that, together with the current therapy, might prevent rabies clinical symptoms, hence supplying time for the organism’s own immune response against the virus that, ultimately, would lead to a cure. It is also noteworthy to comment that, in the present study, viral load was intracerebral, whereas bufotenine treatment was subcutaneous. Moreover, virus titers were much higher (following WHO rabies protocols) than those expected to be consequence of an accident. Therefore, the 40% prevention of symptoms development reported here could be deprecated. If one mimics an accident, in which viruses would typically be injected intramuscularly at a significantly lower titer, the symptoms development prevention could be much higher, thus indicating the need for new studies aiming at the development of this molecule.

### Abbreviations

 CC_50_: cytotoxic concentration; CVS: challenge virus standard; DMSO: dimethyl sulfoxide; ESI-IT-TOF: electrospray-ion trap-time of flight; HE: hematoxylin-eosin; nAChR: nicotinic acetylcholine receptor; NCAM: neural cell adhesion molecule; RABV: rabies viruses; SC: subcutaneous; UFLC: ultra-fast liquid chromatography system. 

## Supplementary material

The following online material is available for this article:

Additional file 1. Percentage of viable N2A cells after treatment with different bufotenine concentrations.

Additional file 2. Onset of symptoms and percent survival of mice infected with CVS 10^-5.32^ (approximately 30LD_50_) and treated with bufotenine 0.63 mg/animal/day.

Additional file 3. Onset of symptoms and percent survival of mice infected with CVS 10^-6.32^ (approximately 3LD_50_) and treated with bufotenine 0.63 mg/animal/day.

Additional file 4. Onset of symptoms and percent survival of mice infected with CVS 10^-6.82^ (approximately 1.35LD_50_) and treated with bufotenine 0.63 mg/animal/day.

Additional file 5. Onset of symptoms and percent survival of mice infected with CVS 10^-7.32^ (approximately 0.3LD_50_) and treated with bufotenine 0.63 mg/animal/day.

Additional file 6. Onset of symptoms and percent survival of mice infected with CVS 10^-6.82^ (approximately 0.3LD_50_) and treated with bufotenine 0.63, 1.05 and 2.1 mg/animal/day.

Additional file 7. Onset of symptoms of mice from bufotenine control groups treated with bufotenine 0.63, 1.05 and 2.1 mg/animal/day.
