# Supplementary material for: Bufotenine, a tryptophan-derived alkaloid, suppresses the symptoms and increases the survival rate of rabies-infected mice: the development of a pharmacological approach for rabies treatment
Source: J Venom Anim Toxins Incl Trop Dis. 2020 Feb 3;26:e20190050. doi: 10.1590/1678-9199-JVATITD-2019-0050 (PMC6996410; doi:10.1590/1678-9199-JVATITD-2019-0050)
Supplement: Additional file 1. [file 1678-9199-jvatitd-26-e20190050-s1.pdf]

Supplementary Material to “Bufotenine, a tryptophan-derived alkaloid, suppress the symptoms and increases the survival rate of rabies-infected mice: the development of a pharmacological approach for rabies treatment”

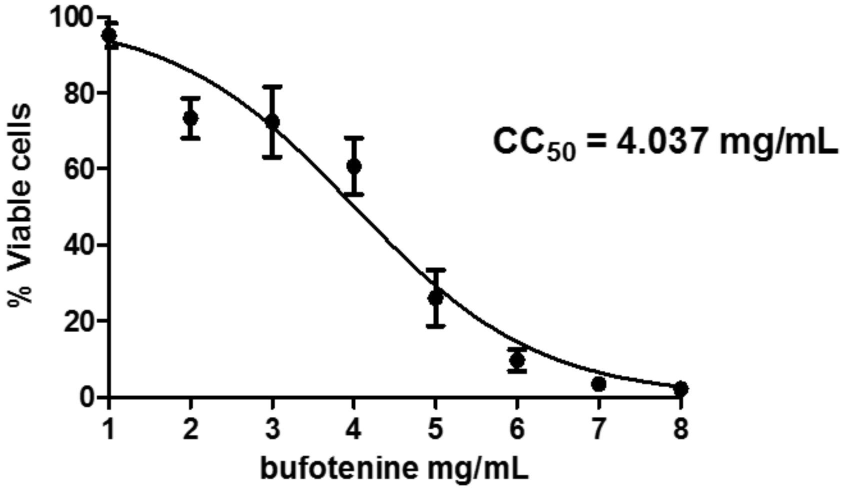

Additional file 1 - Percentage of viable N2A cells after treatment with different bufotenine concentrations. The 50% cytotoxic (CC_{50}) concentration was calculated from concentration-effect curves after linear regression analysis.
