# Supplementary material for: Bufotenine, a tryptophan-derived alkaloid, suppresses the symptoms and increases the survival rate of rabies-infected mice: the development of a pharmacological approach for rabies treatment
Source: J Venom Anim Toxins Incl Trop Dis. 2020 Feb 3;26:e20190050. doi: 10.1590/1678-9199-JVATITD-2019-0050 (PMC6996410; doi:10.1590/1678-9199-JVATITD-2019-0050)
Supplement: Additional file 2. [file 1678-9199-jvatitd-26-e20190050-s2.pdf]

Supplementary Material to "Bufotenine, a tryptophan-derived alkaloid, suppress the symptoms and increases the survival rate of rabies-infected mice: the development of a pharmacological approach for rabies treatment"

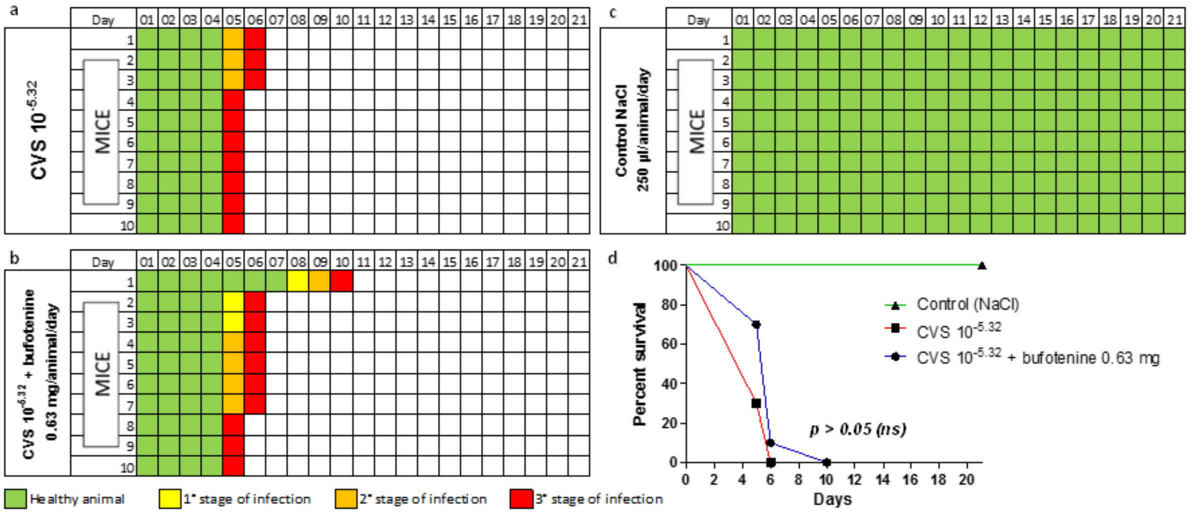

Additional file 2 - Onset of symptoms and percent survival of mice infected with CVS $10^{-5.32}$ (approximately $30LD_{50}$) and treated with bufotenine 0.63 mg/animal/day. **(a)** Rabies control group: onset of symptoms of mice infected with CVS $10^{-5.32}$. **(b)** Treatment group: onset of symptoms of mice infected with CVS $10^{-5.32}$ and treated with bufotenine 0.63 mg/animal/day. **(c)** Control group: NaCl 250 µL/animal/day. **(d)** Percent survival of control group, rabies control group and treatment group. p value summary: $p > 0.05$ (no significant) in both, Log-rank (Mantel-Cox) and Gehan-Breslow-Wilcoxon Tests.
