# Supplementary material for: Bufotenine, a tryptophan-derived alkaloid, suppresses the symptoms and increases the survival rate of rabies-infected mice: the development of a pharmacological approach for rabies treatment
Source: J Venom Anim Toxins Incl Trop Dis. 2020 Feb 3;26:e20190050. doi: 10.1590/1678-9199-JVATITD-2019-0050 (PMC6996410; doi:10.1590/1678-9199-JVATITD-2019-0050)
Supplement: Additional file 6. [file 1678-9199-jvatitd-26-e20190050-s6.pdf]

J Venom Anim Toxins incl Trop Dis, 2020, 26

Supplementary Material to "Bufotenine, a tryptophan-derived alkaloid, suppress the symptoms and increases the survival rate of rabies-infected mice: the development of a pharmacological approach for rabies treatment"

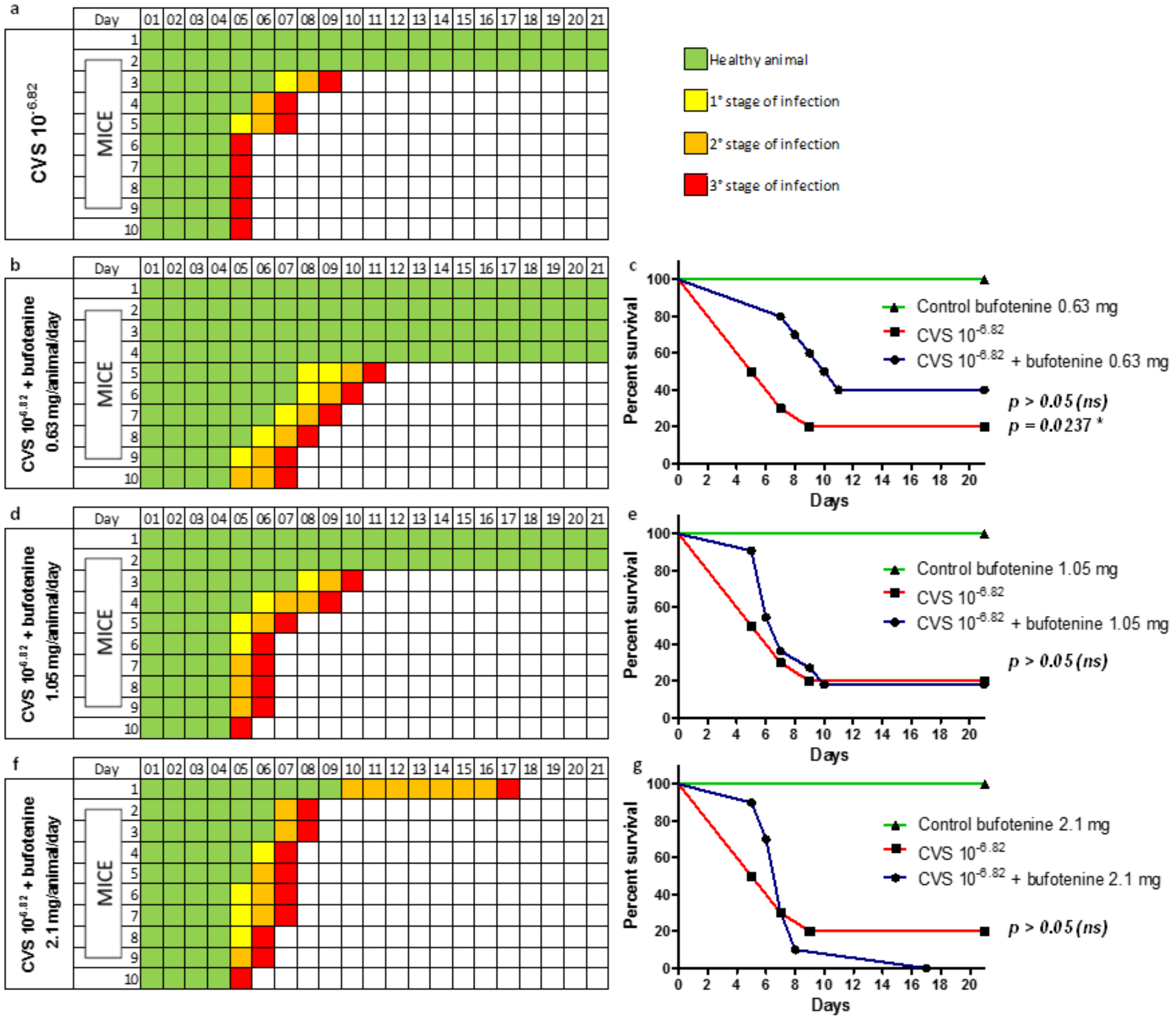

Additional file 6 - Onset of symptoms and percent survival of mice infected with CVS $10^{-6.82}$ (approximately $0.3LD_{50}$) and treated with bufotenine 0.63, 1.05 and 2.1 mg/animal/day. (a) Rabies control group: onset of symptoms of mice infected with CVS $10^{-6.82}$. **(b)** Treatment group: onset of symptoms of mice infected with CVS $10^{-6.82}$ and treated with bufotenine 0.63 mg/animal/day. **(c)** Percent survival of control group, rabies control group and treatment group with 0.63 mg/animal/day. **(d)** Treatment group: onset of symptoms of mice infected with CVS $10^{-6.82}$ and treated with bufotenine 1.05 mg/animal/day. **(e)** Percent survival of control group, rabies control group and treatment group with 1.05 mg/animal/day. **(f)** Treatment group: onset of symptoms of mice infected with CVS $10^{-6.82}$ and treated with bufotenine 2.1 mg/animal/day. **(g)** Percent survival of control group, rabies control group and treatment group with 2.1 mg/animal/day. p value summary: $*p \leq 0.05$ in Gehan-Breslow-Wilcoxon Test, $p > 0.05$ (no significant) in Log-rank (Mantel-Cox), Gehan-Breslow-Wilcoxon or both Tests.
