# Supplementary material for: Bufotenine, a tryptophan-derived alkaloid, suppresses the symptoms and increases the survival rate of rabies-infected mice: the development of a pharmacological approach for rabies treatment
Source: J Venom Anim Toxins Incl Trop Dis. 2020 Feb 3;26:e20190050. doi: 10.1590/1678-9199-JVATITD-2019-0050 (PMC6996410; doi:10.1590/1678-9199-JVATITD-2019-0050)
Supplement: Additional file 7. [file 1678-9199-jvatitd-26-e20190050-s7.pdf]

Supplementary Material to "Bufotenine, a tryptophan-derived alkaloid, suppress the symptoms and increases the survival rate of rabies-infected mice: the development of a pharmacological approach for rabies treatment"

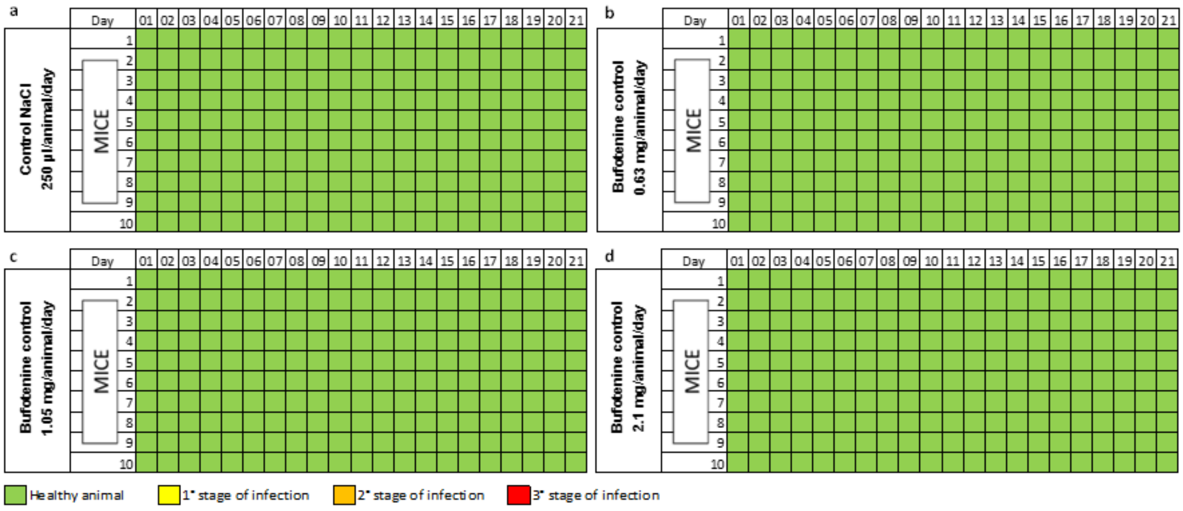

Additional file 7 - Onset of symptoms of mice from bufotenine control groups treated with bufotenine 0.63, 1.05 and 2.1 mg/animal/day. (a) Control group: NaCl 250 µL/animal/day. **(b)** Bufotenine control group: treated with bufotenine 0.63 mg/animal/day. **(c)** Bufotenine control group: treated with bufotenine 1.05 mg/animal/day. **(d)** Bufotenine control group: treated with bufotenine 2.1 mg/animal/day.
